# Revisiting Louis Fry’s spiritual leadership model in confessional school teachers using structural equation modeling (SEM)

**DOI:** 10.1371/journal.pone.0299671

**Published:** 2024-09-17

**Authors:** Lorena Martinez-Soto, Flor Ontiveros Ramírez, Iván Dario Toro Jaramillo, Nelly Rosario Moreno-Leyva

**Affiliations:** 1 Faculty of Administrative and Accounting Sciences, Corporación Universitaria Adventista, Medellín, Antioquia, Colombia; 2 Faculty of Business and Legal Sciences, Universidad de Montemorelos, Montemorelos, Nuevo Leon, Mexico; 3 School of Theology, Universidad Pontificia Bolivariana, Medellín, Antioquia, Colombia; 4 Professional School of Accounting, Faculty of Business Sciences, Universidad Peruana Unión, Juliaca, Puno, Peru; Universiti Pertahanan Nasional Malaysia, MALAYSIA

## Abstract

This paper examines the construct validity of the spiritual leadership model proposed by (Fry et al. 2005). The analysis focused on examining the relationships proposed by the model through CFA and structural equation modeling (SEM). A confirmatory factor analysis indicated the SL scale provides acceptable reliability and convergent validity indexes; however, it did not achieve discriminant validity. Model convergence was obtained using MLR (Robust Maximum Likelihood) methods. However, when the robustness indices were analyzed, it was found that some obtained acceptable results and others were deficient, so that an acceptable model fit was not achieved. Regarding the relationship between the hypotheses, it was found that they were significant in all cases except for the reciprocal relationship between vision and altruistic love. In light of this finding, alternative models were developed that also failed to yield significant results. The theoretical and methodological discussion focuses on the relationships of Fry’s model and addresses the need to review its causal nature, considering recursive and non-recursive aspects.

## Introduction

Workplace Spirituality (WS) is an area of research that has roots in Philosophy and Theology and has grown exponentially in organizations due to its ability to solve problems [1–4]. At the beginning of this century, the first studies were published showing the links between spirituality and the world of work [1–4]. Topics such as the influence of spirituality on employee well-being [5], organizational commitment [6], motivation [7], equitable payment, job satisfaction [8], building a shared vision [9], emotional exhaustion [10], and organizational performance [11], are some of the issues managers have sought to resolve through WS.

In WS, leadership styles based on spirituality are among the best management tools for obtaining positive organizational results. This is because they promote work environments focused on well-being and meeting the spiritual needs of their employees. With an approach oriented to the construction of meaning and a sense of purpose, spiritual leadership (SL) is particularly effective in creating workplaces characterized by bonds beyond the contractual relationship.

Although many studies confirm the positive impact of spirituality on business organizations, few demonstrate its effectiveness in religious organizations. This is possible because Theology studies this type of phenomenon most directly, and additionally, it is taken for granted that spirituality is an identity characteristic of confessional organizations [12–14].

Education, at its different levels and modalities, has been one of the most important areas of development and expansion of religious organizations. In the case of Protestant denominations, the construction of schools, seminaries and universities is a way of teaching religious principles and values.

In Latin American countries, a number of social issues have emerged, including violence, drug trafficking, high levels of unemployment, poverty, and marginalization [10, 15, 16]. These challenges have led to the emergence of denominational schools as a plausible alternative for educating new generations in moral principles and values that contribute to social transformation. The number of studies exploring the role of religion and spirituality as a response to the crisis of values is growing [17–19]. Such studies have shown that denominational schools have a positive impact on reducing rates of school violence [20], drug use [21], bullying [22], and racial discrimination [23].

As in business organizations, the SL or WS models are highly useful in the administrative management of confessional schools. In this context, the objective of this study is to address the following research question (RQ): What is the validity and reliability of the SL scale according to the opinion of teachers in Adventist primary and secondary schools in Colombia?

To answer this question, a CFA was carried out to determine the degree of significance of the relationships proposed in the Model, and an SEM analysis was conducted to identify the robustness of the Model.

### Theoretical framework

The SL theory, proposed by [3, 24–26], is based on the perspective of the organization as a space of social interaction that seeks to promote the spiritual well-being of employees through spiritually rooted leadership.

This approach aims to create an organizational culture in which the organization and the employee work together to achieve a vision that exceeds corporate limits [9, 27–29]. The transcendent component of the vision seeks to motivate employees intrinsically, so it tries to respond to your needs for purpose and meaning. This requires creating an inspiring vision whose narrative promotes a work environment where beliefs and practices continually reinforce motivation to achieve the vision.

For [30], SL is not only a leadership style but a theoretical model that can explain the leader’s ability to build a transcendent vision, maintain faith and hope in that vision, and practice altruistic behaviors based on spiritual values that promote an environment of spiritual well-being in which people develop a sense of purpose and meaning [5, 27, 31, 32].

The variables that make up the SL model of [30] are:

Vision: it is the expression of an ideal to achieve that transcends corporate limits and is projected in the long term. A transcendent vision must be challenging, desirable, and possess inspiring potential that persuades employees to identify with it and commit to joining efforts with the organization to achieve it [3, 24, 25].

Hope-Faith: These are the behaviors and attitudes that demonstrate the firm conviction that the vision will be fulfilled. This implies actions and attitudes on the part of the leader that must be consistent with the system of spiritual values that support the vision. In this way, coherence between what is said and what is done will reinforce the construction of trust between the leader and employees [31, 33].

Altruistic Love: Refers to the attitudes and behaviors that the leader must demonstrate in his or her daily activities. His beliefs should be evidenced in his sense of ongoing compassion, empathy, humility, and service [25, 26, 34].

Vocation/Calling: It is the need that human beings have to feel that they are contributing to the achievement of a purpose that goes beyond a contractual relationship. The literature shows that people not only seek to practice their profession successfully but also need to experience that their work provides social value and contributes to other people having a better quality of life [29, 30, 35].

**Fig 1:**
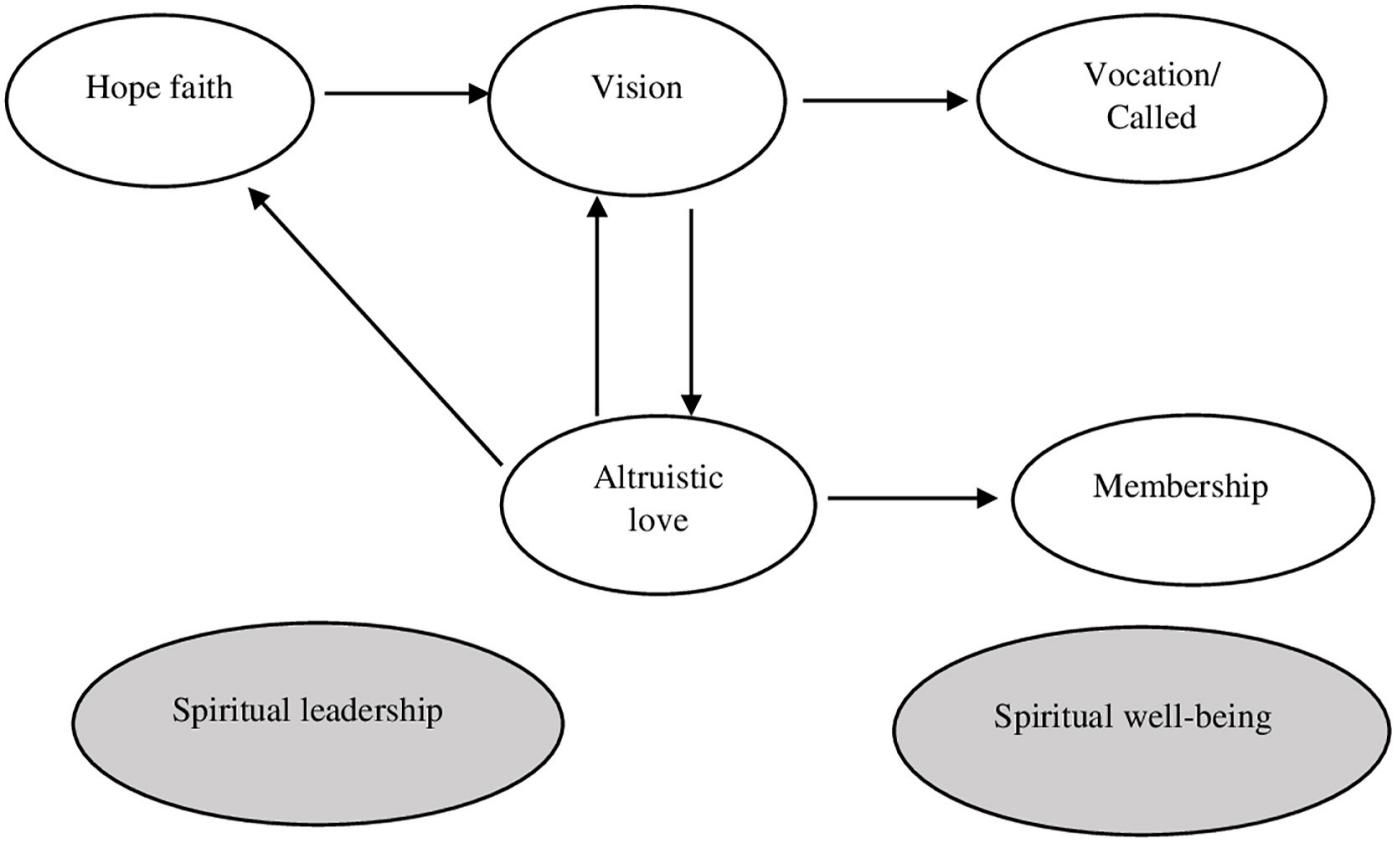
Fry’s spiritual leadership model.

Membership: It is the human being’s need to belong. The possibility of feeling that you are part of a community that recognizes and appreciates you. Literature maintains that human beings have an inherent need for connection and social valuation that leads them to look for work spaces with which they feel identified [36–38] (see Fig 1).

According to [30], the relationships between these five variables give rise to a model made up of six (6) causal relationships or hypotheses of the study: **H1**: The variable hope/faith (ef) positively influences vision (vision). An inspiring vision will influence the development of attitudes and behaviors based on spiritual values consistent with the vision. This conviction must manifest itself in the practice of spiritual values that reinforce belief in the vision and generate a collective identity in which the same beliefs and values are shared. [34] conducted a study in the Korean cultural context and obtained results similar to those of [30], reinforcing the Model’s validity. In this case, the causal relationship between hope/faith in vision was (*β* = 0.500, *p* < 0.01).

**H2**: The vision variable (vision) positively influences altruistic love (aa). The practice of altruistic behaviors based on spiritual principles and values shared by the group, which in turn are consistent with the vision, reinforces the hope/faith that the vision will be fulfilled. Again, the study of [34] obtained similar results to the SL model in what corresponds to the relationship between the variables vision and altruistic love (*β* = 0.49 with value *p* < 0.01).

**H3**: The variable altruistic love (aa) positively influences hope/faith (ef). The altruistic love variable not only influences hope-faith, but also strengthens belief in vision. According to [30], this occurs when the organization fosters spiritual values reflected in altruistic behavior that reduces prejudices, worries and selfishness. The study by [39], confirms this hypothesis by finding that altruistic love generates trust between people, acting as a source of hope and faith to complete the work.

**H4**: The variable altruistic love (aa) positively influences vision (vision). The practice of behaviors guided by moral and spiritual values such as cooperation, compassion, empathy, service and altruism, lead to the creation of a work environment that reinforces the vision. In this way, the coherence between beliefs and actions sends the employee a consistent message that strengthens the spiritual environment of the organization.

**H5**: The vision variable (vision) positively influences the vocation (voca). The literature shows that people not only seek to practice their profession successfully but also need to connect with a cause that allows them to experience that their work provides social value and contributes to a better quality of life for others. The study of [40] supports this hypothesis by obtaining positive results and significant relationships between the constructs. Consistent with the findings, all coefficients were significant (*p* < 0.01), and the results of the structural Model indicated that a vision based on spirituality has a positive effect on vocation/calling. (in the Polish sample (*β* = 0.97, *p* < 0.01), and for the Bhutanese sample (*β* = 0.67, *p* < 0.01). Additionally, [41] also obtained positive and significant results between vision and vocation. Their study was conducted with hotel employees from various cities in China, and the hypothesis test confirmed a positive and significant causal relationship (*β* = 0.21, *p* < 0.001).

**H6**: The variable altruistic love (aa) positively influences membership (belonging). Literature maintains that human beings have an inherent need for social connection and value. Therefore, when leaders and employees share the same beliefs, practices, values and ideals, the employee experiences a sense of spiritual connection that increases their sense of belonging with the organization and with their colleagues. This relationship between variables is supported by the study by [42] who used the [30] to explore the relationship between spiritual leadership and organizational commitment in teachers from primary and secondary schools in Hong Kong. The study revealed that the SL perceived by teachers is a significantly positive predictor of calling, with *β* = 0.528, *p* < /0.001 and membership *β* = 0.704, *p* <.001.

## Materials and methods

### Participants

The data were collected via an online questionnaire hosted by Google, which was linked to an Excel spreadsheet. The majority of participants were female (53.2%), and the most common age range was between 31 and 40 years (37.5%), followed by the 20–30 age group (34.1%). In terms of educational background, 51.2% of the teachers had obtained an undergraduate degree, while 28.8% had completed a graduate degree. In terms of seniority, the majority of participants had between one and three years of seniority (29.8%), followed by those with between four and six years (26.4%). In terms of religious affiliation, 91% of the professors reported being Adventists. With regard to the type of contract, the majority of contracts were one-year fixed-term contracts (65.6%), with indefinite-term contracts representing the second most common type (16.1%).

### Ethics statement

Participants’ confidentiality and privacy were maintained throughout the research. Informed consent was obtained via a “confidentiality agreement” section at the beginning of the Google questionnaire, allowing voluntary participation. The Adventist University Corporation Ethics Committee approved the study on July 10, 2023, based on its relevance, originality, ethical integrity, and scientific rigor (minute number 003, 2023).

### Measurement instrument

The measurement instrument used was the SL model scale from [3]. This scale is composed of five latent variables: vision (4 statements), hope/faith (4 statements), altruistic love (5 statements), vocation (4 statements) and membership (4 statements). The SL scale is composed of twenty-one (21) statements with five-point Likert-type response options, ranging from “strongly disagree” = 1 to “strongly agree” = 5.

Since the original scale was in a different language, the back-translation process from English to Spanish followed the guidelines of Bracken [43, 44]. This process, necessary to guarantee the instrument’s content validity, involved translating, adapting, and validating it. The result was a final version in Spanish, which was subjected to a pilot test with a group of teachers and experts (N = 10) to adjust aspects that ensured the clarity, relevance and understanding in the local context.

### Data collection procedure

The data collection process was conducted in two stages. The initial stage began with a formal invitation to participate in the study. The message provided a detailed explanation of the purpose of the survey and the target population, along with a link to the Google form that included the request for authorization through informed consent and the instrument. Subsequently, a telephone follow-up was conducted with the objective of motivating teachers participation. In the second stage, an additional sampling was conducted, thus completing a total of 299 respondents (124 in the first and 175 in the second). The target population consisted of thirteen (13) Adventist schools. The data were collected between September 4, 2023, and March 12, 2024. Of the 347 individuals in the target population, 299 responded to the questionnaire, representing an 86% response rate. In this instance, it was determined that although the sample was non-probabilistic, the percentage of participants allowed for the sample to be considered representative and generalizable to the target population.

### Sample size

The sample size selected was power analysis *post hoc* because the sample had already been taken at the time of the analysis. It was chosen RMSEA as the value to use to determine the power of the test taking as appropriate values ≤ 0.05. When doing the calculation, it was found that the power of the test exceeds 80%; therefore, it is an appropriate sample for the type of analysis that will be performed. The sample calculation was made taking into account the most recent publications for modeling based on structural equations [45]. Although an analysis post hoc [46] is not necessary to support the sample size, it does help determine that the instrument has sufficient power to validate the construct (see Table 1).

**Table 1:** Complete model reliability.

Parameter	Value
F0	0.732500
RMSEA	0.050000
Mc	0.693329
Df	293
Num observations	299
NCP	218.2850
Critical Chi-Square	333.9218
Alpha	0.050000
Beta	8.243275e-08
Power (1-Beta)	> 0.9999
Implied Alpha/Beta Ratio	6.065550e+05

### Data processing

To process the data, tests were carried out to verify and adjust the proposed models. Skewness and kurtosis were used, among other tests, taking into account the assumption of univariate and multivariate normality through the Shapiro-Wilk and multivariate Mardia tests. Because it is common for the assumptions of the maximum likelihood fit method to not be met; robust maximum likelihood methods of the R *lavaan* package were used, such as MLR (Maximum Likelihood Robust) equivalent to the Yuan-Bentler correction and the MLM (Adjusted Scaled Mean Maximum Likelihood) method or [47] correction.

### Robustness indices

The indices used to evaluate the robustness of the Model fit in the SEM were *χ*^2^ (Chi-square); GFI (Goodness-of-fit index); NFI (Normed Fit Index); IFI (Incremental Fit Index); CFI (Comparative Fix Index); RMSEA (Root Mean Square Error of Approximation). [48–50] explain the Model’s goodness-of-fit measures. It is well known that c2 statistics are almost always large and statistically significant for complex models. Generally, GFI, CFI and TLI values equal to or above 0.95 were considered an “optimal” fit [50]. In contrast, values equal to or above 0.90 were considered “adequate” fit. Values less than 0.90 were considered an “poor” fit. RMSEA fit indices less than or equal to 0.05 were denoted as an “adequate” fit.

### Statistical analysis procedure

The validity and reliability of the model were tested through a CFA and an SEM analysis of the SL model proposed by [51], which included all the suggested relationships between variables.

- **CFA procedure** The *lavaan* package and the function *cfa()* were used to adjust the CFA. Initially, whether the items statistically represented the defined latent variables was verified. Once the results were obtained, discriminant validity was carried out.
- **SEM analysis procedure** To adjust the SEM, the coefficients of the hypotheses raised were estimated using the function *sem()* of the *lavaan* package. It was considered to use the methodology robust maximum likelihood in case of not obtaining model convergence using the standard procedure.

### Instrument validity and reliability

Validity and reliability were calculated using Cronbach’s Alpha. (ALPHA); Composite Reliability (CR) and Average Variance Extracted (AVE).

### Software and tools

The R statistical language version 4.4.0 was used with the R-Studio [52] version 2023.12.0+369 interface. Packages complemented it was *lavaan* [53], which uses the statistical test Sattora-Bentler chi-square for the estimation of the parameters of the confirmatory analysis, *semTools* [54] complementary tools for the estimation of confirmatory analysis of structural equation modeling, *tidyverse* [55] database management and debugging, *lavaanPlot* [56], for the graphing of the resulting models, *rempsyc* [57] generation of tables from the results of structural equations, *psych* [58] calculation of psychometric parameters, *gtsummary* [59] to obtain summary tables, *Hmisc* [60] complementary functions for structural equation modeling, *tidySEM* [61], *semPower* [45], ad-hoc validity estimation of the sample size and flexible obtaining of tables that allow manipulation in other formats [62].

## Results

### CFA modeling

**Fig 2:**
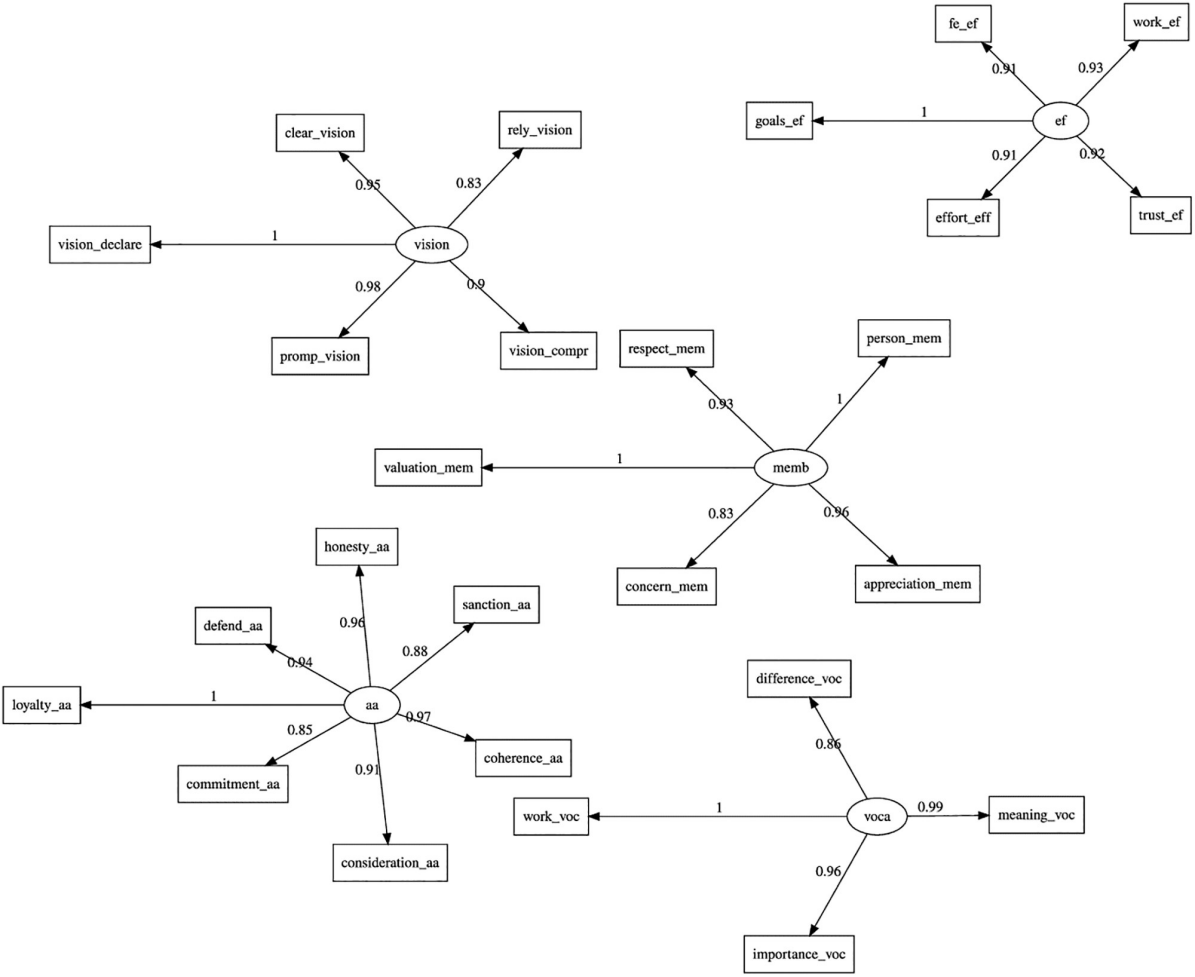
CFA full model.

As mentioned earlier, the objective of the CFA modeling was to ascertain whether the items statistically represented the latent variables. It is important to note that, according to the literature, the primary objective of CFA is to verify that items are associated with their latent variables and to demonstrate that these variables are independent of each other. In this case, CFA was not intended to meet the second condition, as the [30] model is causal in nature (see Fig 2 and Table 2).

**Table 2:** CFA parameter coefficients complete model.

Latent Variables	Estimate	Std.Err	z-value	P(>|z|)	Std.lv	Std.all
**vision**						
vision_dec1are	1.000				0.829	0.830
promp_vision	0.978	0.088	11.079	0.000	0.811	0.812
vision_compr	0.904	0.081	11.122	0.000	0.749	0.751
rely_vision	0.834	0.079	10.621	0.000	0.691	0.692
clear_vision	0.955	0.091	10.478	0.000	0.791	0.793
**ef**						
goals_ef	1.000				0.809	0.810
effort_eff	0.907	0.084	10.743	0.000	0.734	0.735
trust_ef	0.921	0.088	10.514	0.000	0.745	0.746
work_ef	0.930	0.085	10.961	0.000	0.752	0.754
fe_ef	0.910	0.113	8.079	0.000	0.736	0.737
**aa**						
loyalty_aa	1.000				0.777	0.779
commitment_aa	0.855	0.093	9.208	0.000	0.664	0.665
consideration_	0.912	0.080	11.431	0.000	0.709	0.710
coherence_aa	0.971	0.085	11.400	0.000	0.755	0.756
sanction_aa	0.879	0.086	10.188	0.000	0.683	0.684
honesty_aa	0.958	0.074	12.870	0.000	0.745	0.746
defend_aa	0.936	0.072	13.040	0.000	0.728	0.729
**voca**						
work_voc	1.000				0.849	0.851
importance_voc	0.957	0.091	10.551	0.000	0.813	0.814
meaning_voc	0.988	0.073	13.486	0.000	0.839	0.841
difference_voc	0.860	0.098	8.734	0.000	0.731	0.732
**memb**						
valuation_mem	1.000				0.822	0.824
concern_mem	0.827	0.074	11.191	0.000	0.680	0.681
appreciation_mem	0.965	0.072	13.482	0.000	0.793	0.795
person_mem	0.100	0.077	12.969	0.000	0.822	0.824
respect_mem	0.934	0.090	10.370	0.000	0.768	0.769

#### Complete CFA model FIT

The complete confirmatory factor analysis model under consideration was analyzed according to the following goodness-of-fit indices: *χ*^2^(289) = 336.974, *p* < 0.000; normalized chi-squared (*χ*^2^/*df* = 1.46); GFI = 0.819; scaled RMSEA = 0.024; NFI = 0.869; CFI = 0.979; and IFI = 0.979. An overall analysis of the indices shows that 4 out of the 7 goodness-of-fit indices were achieved. Therefore, it was concluded that although these results did not invalidate the model, they did reveal certain weaknesses, which are examined in the SEM analysis [34].

#### Complete instrument’s validity and reliability

To assess the validity and reliability of the instrument, the results were analyzed using Cronbach’s Alpha and composite reliability (CR) coefficients. Additionally, the Average Variance Extracted (AVE) and the correlations between variables were calculated, with the square root of the AVE included in the diagonal of the correlation matrix. Convergent validity was considered acceptable if it exceeded the criterion of >0.50. Discriminant validity was considered adequate if the value of the square root of the AVE was greater than the absolute value of each of the correlations of the other constructs in the model (see Table 3).

**Table 3:** Correlations between constructs for the scale based on the current sample of the complete model.

Factor	Alpha	CR	AVE	Vision	EF	AA	Voca	Memb
Vision	0.882	0.884	0.604	0.777				
EF	0.869	0.870	0.573	0.900	0.757			
AA	0.885	0.886	0.526	0.783	0.820	0.725		
Voca	0.883	0.884	0.657	0.827	0.956	0.686	0.811	
Memb	0.884	0.886	0.609	0.780	0.799	0.933	0.760	0.780

As can be seen, the reliability of all constructs exceeds the threshold of 0.70 [34], as well as the composite reliability (CR), which should be above 0.70 [34]. Therefore, these results show the reliability of the instrument and the suitability of the indicators for the empirical explanation of the latent constructs.

Convergent validity was assessed using the average variance extracted (AVE) of each construct according to previously established criteria. As can be seen in Table 3, all constructs scored above 0.50 and were therefore considered to have convergent validity.

**Fig 3:**
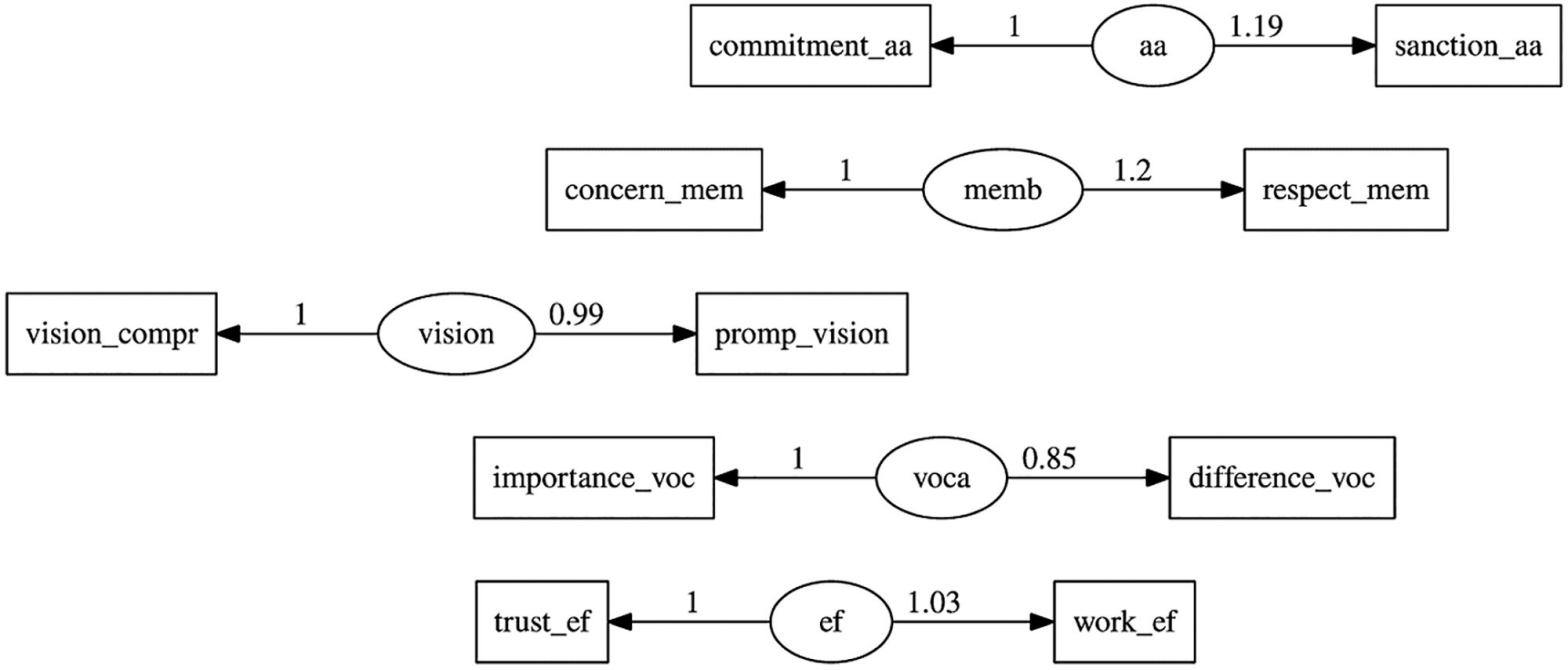
CFA refined model.

Regarding discriminant validity, when analyzing the results of the square roots of the AVE, it was found that the complete model did not meet the [34] criterion, indicating the presence of discriminant validity. Therefore, the analysis proposed by [34] to assess discriminant validity was carried out. This analysis was conducted through an iterative process by removing the items with the lowest factor loadings until a better fit was achieved. The items removed were: effort_ef, goals_ef, fe_ef, vision_declare, rely_vision, clear_vision, meaning_voca, work_voca, consideration_aa, coherence_aa, loyalty_aa, honesty_aa, defend_aa, appreciation_mem, valuation_mm, and person_mm (see Table 4 and Fig 3).

**Table 4:** Parameter coefficients CFA refined model.

Latent Variables:	Estimate	Std.Err	z-value	P(>|z|)	Std.lv	Std.all
vision =						
vision_compr	1				0.738	0.778
promp_vision	0.987	0.111	8.894	0	0.729	0.807
ef =						
trust_ef	1				0.675	0.796
work_ef	1.033	0.099	10.466	0	0.697	0.787
aa =						
commitment_aa	1				0.54	0.642
sanction_aa	1.194	0.192	6.208	0	0.644	0.677
voca =						
importance_voc	1				0.722	0.81
difference_voc	0.855	0.091	9.434	0	0.617	0.757
memb =						
concern_mem	1				0.609	0.678
respect_mem	1.202	0.146	8.237	0	0.733	0.807

#### Refined CFA model FIT

The cleaned confirmatory factor analysis model under consideration was analyzed according to the following goodness of fit indices: *χ*^2^ (25) = 53.087, *p* < 0.000; normalized chi-squared (*χ*^2^/df) = 2.123; GFI = 0.994; scaled RMSEA = 0.061, NFI = 0.929, CFI = 0.960, and IFI = 0.961 [50]. When analyzing the indices holistically, it was observed that as items were eliminated, the model fit improved and 5 out of the 7 goodness of fit indices were achieved. Therefore, it was concluded that although these results did not invalidate the model, they did show certain weaknesses.

#### Refined instrument’s validity and reliability

Once the nine items with the lowest factor loadings had been eliminated, the analysis was performed again with the cleaned model. The results demonstrated that the lack of discriminant validity persisted (see Table 5).

**Table 5:** Correlations between constructs for the scale based on the current sample of the refined model.

Factor	ALPHA	CR	AVE	Vision	EF	AA	Voca	Memb
Vision	0.771	0.771	0.628	0.792				
EF	0.770	0.770	0.626	0.857	0.791			
AA	0.603	0.607	0.438	0.864	0.767	0.662		
Voca	0.758	0.763	0.618	0.839	0.943	0.745	0.786	
Memb	0.707	0.713	0.557	0.610	0.752	0.839	0.742	0.746

It is important to highlight that the researchers did not consider the option of conducting an exploratory factor analysis (EFA) because the aim of this research is to use the SL model as part of a larger research project. For this reason, we insisted on validating the original model, also because of the considerable body of previous studies that supported the model’s validity.

### SEM analysis

In this analysis, the structural model was applied so that once adjusted, the formulated hypotheses could be validated. The estimation of SEM parameters was carried out using the Maximum Robust Likelihood (MLR) method equivalent to the Yuan-Bentler correction and the MLM method (Maximum Likelihood of Scaled Adjusted Mean) or the Satorra-Bentler correction (2001) due to the non-normal distribution of the items. To interpret the goodness-of-fit indices, the parameters of the scaled variables proposed by [50] were employed.

#### Complete model

The fit of the complete model was assessed and the following results were obtained: *χ*^2^ (292) = 467.891, *p* < 0.000; normalized chi-square *χ*^2^/df = 1.602 < 3; GFI = 0.803; RMSEA = 0.045, NFI = 0.843, CFI = 0.934, IFI = 0.934. When estimating the relationships between variables, it was found that H1, H3, H5, and H6 showed positive and significant results. However, the reciprocal relationship between vision and altruistic love was not significant, with values of -0.044 in the relationship from vision to altruistic love (H2) and 0.076 in the relationship from altruistic love to vision (H4) (see Table 6).

**Table 6:** Complete model regression coefficients.

	Estimate	Std.Err	z-value	P(>|z|)	Std.lv	Std.all
vision ∼ ef	0.903	0.145	6.211	0	0.906	0.906
vision ∼ aa	0.076	0.109	0.699	0.485	0.073	0.073
ef ∼ aa	0.872	0.122	7.170	0	0.835	0.835
aa ∼ vision	-0.044	0.108	-0.405	0.685	-0.046	-0.046
voca ∼ vision	0.932	0.105	8.868	0	0.898	0.898
memb ∼ aa	1.009	0.079	12.811	0	0.944	0.944

Given these results, possible explanations were sought. One of these was to analyse the recursive and non-recursive aspects of the model, which according to [30] is considered a causal model. Theoretically, this implies that the relationships between variables should be unidirectional; however, when looking at the SL model, is found to contain dual causality relationships and feedback loops between variables that behave according to the parameters of a loop.

With this in mind, two alternative models were proposed, each expressing one of the relationships that make up reciprocity. It is important to clarify that the purpose of this exercise was not to improve discriminant validity, but rather to seek possible explanations for the results obtained.

With the alternative models (recursive and non-recursive) we tried to represent the relationships of the SL model in two different structures, because each directionality implies different relationships between variables.

In this sense, it is considered important to highlight the argument of [63], which explains that non-recursive models tend to be unidentifiable and cannot be estimated. This is because these models may hide a theoretical weakness in that they are unable to identify the causal relationship between the two variables.

They proposed: 1) a recursive model in which altruistic love influences vision, and 2) a non-recursive model in which vision influences altruistic love.

#### Recursive model

**Fig 4:**
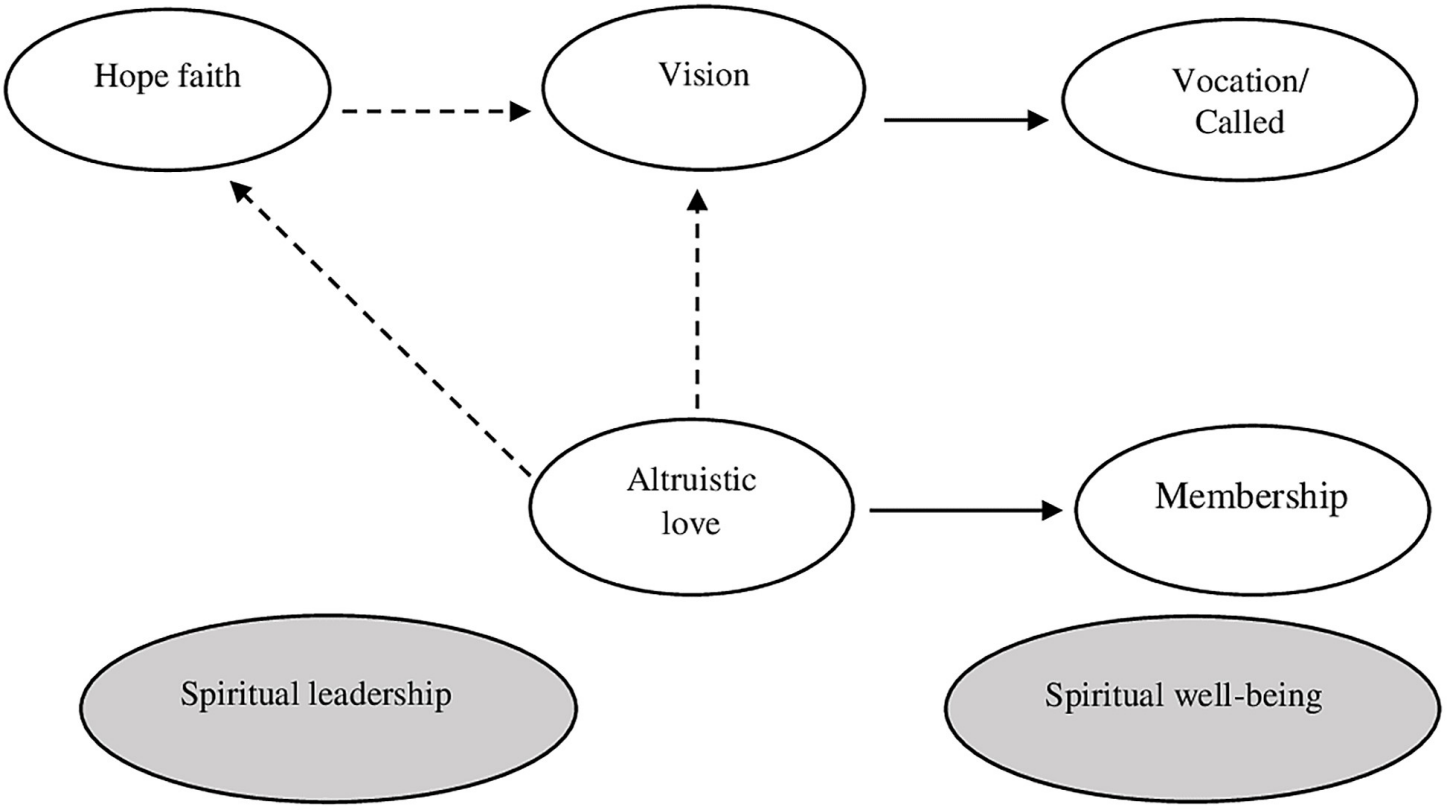
Recursive model.

According to [63], these models do not show reciprocity or loops. In this case, the relationships are: altruistic love explains vision and hope/faith; hope/faith explains vision; vision explains calling; and altruistic love explains membership. Note that in this case altruistic love is not explained by any variable (see Fig 4).

We proceeded to evaluate the fit of the recursive model, obtaining the following results: *χ*^2^ (293) = 369.914, *p* = 0.002; normalized chi-square *χ*^2^/df = 1.019 < 1.262; GFI = 0.803; RMSEA = 0.030, NFI = 0.856, CFI = 0.966, IFI = 0.966. When estimating the relationships between the latent variables proposed in the SL model, it was found that H1, H3, H5, and H6 showed positive and significant results; however, the relationship between altruistic love and vision (H4) was not significant (0.063) (see Table 7).

**Table 7:** Recursive model regression coefficients.

	Estimate	Std.Err	z-value	P(>|z|)	Std.lv	Std.all
ef ∼ aa	0.85	0.103	8.235	0	0.822	0.822
vision ∼ ef	0.795	0.111	7.185	0	0.905	0.905
vision ∼ aa	0.063	0.076	0.837	0.403	0.070	0.070
voca ∼ vision	1.022	0.107	9.528	0	0.898	0.898
memb ∼ aa	1.062	0.081	13.134	0	0.944	0.944

#### Non recursive model

**Fig 5:**
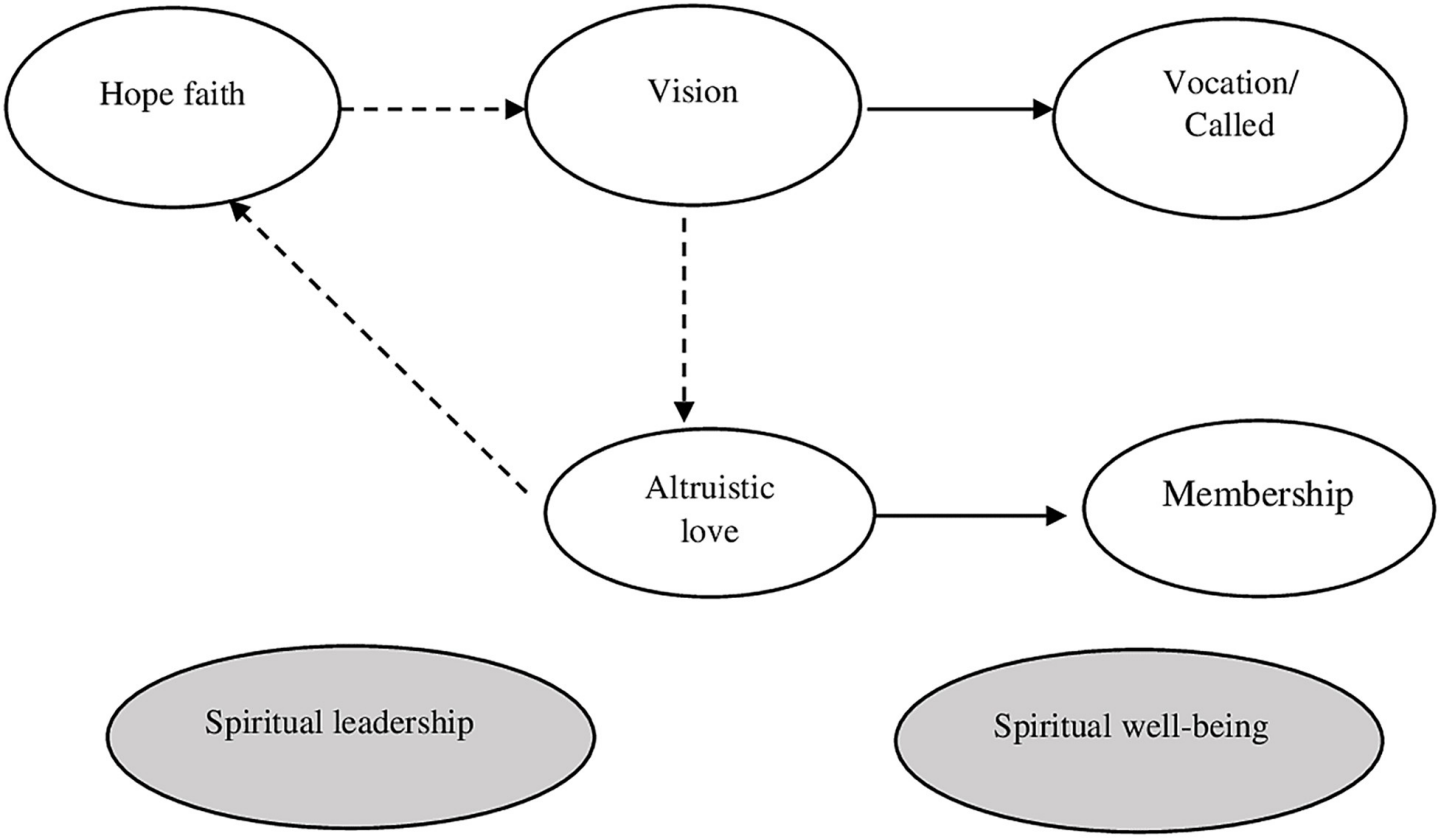
Non recursive model.

According to [63], these models show reciprocity and loops. In this case, the relationships between the variables are reciprocal because vision explains altruistic love, altruistic love explains hope/faith, and finally hope/faith explains vision; vision explains vocation; and altruistic love explains membership (see Fig 5).

The fit of the non-recursive model was assessed and the following results were obtained: *χ*^2^ (269) = 431.532, *p* < 0.000; normalized chi-square *χ*^2^/df = 1.604 < 3; GFI = 0.813; RMSEA = 0.045, NFI = 0.847, CFI = 0.936, IFI = 0.936. When estimating the relationships between the latent variables proposed in the SL model, it was found that H1, H5, H6 showed positive and significant results; however, the relationship between vision and altruistic love (H2) was not significant (0.210) and the relationship between altruistic love and hope/faith (H3) was also not significant (0.774) (see Table 8).

**Table 8:** Non recursive model regression coefficients.

	Estimate	Std.Err	z-value	P(>|z|)	Std.lv	Std.all
ef ∼ aa	0.774	0.753	1.028	0.304	0.755	0.755
aa ∼ vision	0.210	1.898	0.111	0.912	0.186	0.186
vision ∼ ef	0.843	0.115	7.34	0	0.977	0.977
voca ∼ vision	1.074	0.121	8.873	0	0.920	0.920
memb ∼ aa	1.066	0.083	12.886	0	0.945	0.945

Table 9 presents the outcomes of the model adjustments, enabling a comparative analysis of the results. In the three models, four of the seven indexes yielded satisfactory results.

**Table 9:** Model fit statistics (n = 292).

Model	X2	X2/df	GFI	NFI	CFI	IFI	RMSEA
Complete model	467.891, *p* < 0.000	1.602	0.803	0.843	0.934	0.934	0.045
Recursive model	369.914, *p* < 0.002	1.262	0.803	0.856	0.966	0.966	0.030
Non-recursive model	431.532, *p* < 0.000	1.604	0.813	0.847	0.936	0.936	0.045
Criterion	-	< 3	> 0.90	> 0.90	> 0.90	> 0.90	< 0.08

Regarding the hypotheses, when the results of the SEM analysis are analyzed holistically, it is found that there are non-significant relationships in the three models, namely in the original version of the SL model by [30] (called the complete model), H2 and H4 were shown to be non-significant. In the first alternative version, called the recursive model, H4 was not found to be significant. In the second alternative version, called the non-recursive model, H2 and H3 were not found to be significant. Note that in all cases the reciprocal relationship between vision and altruistic love was not found to be significant (see Table 10).

**Table 10:** Standardized regression coefficients of the models.

Hypothesis	Complete model	Recursive model	Non-recursive model	Significance
H1 ef—vision	0.906***	0.905***	0.977***	S
H2 vision—aa	-0.046	NA	0.186	NS
H3 aa—ef	0.835***	0.822***	0.755	S/NS
H4 aa—vision	0.0730	0.070	NA	NS
H5 vision—voca	0.898***	0.898***	0.920***	S
H6 aa—memb	0.944***	0.944***	0.945***	S

Note: *** degree of significance. S = significant relationship, NS = non-significant relationship. *p <.05, **p <.01, ***p <.001

## Discussion

SL emerges as a leadership style derived from transformational leadership and as a new alternative that places the inner life of the human being at the center of organizational management. The publications by [64–66] illustrate the long trajectory of the transition from transformational leadership to a theoretical model of spiritual leadership. Common aspects include the role of the leader (vision, values, attitudes, behaviors), their connection to the intrinsic motivations of followers (identity, values, aspirations, and needs), and the importance of achieving favorable organizational outcomes.

In this context, the objective of this research was to evaluate the construct validity of the SL model in the context of teachers in primary and secondary confessional schools in Colombia using CFA and SEM analysis.

### Discussion of Confirmatory Factor Analysis (CFA)

According to the CFA results, while convergent validity and instrument reliability were achieved, discriminant validity was not. Discriminant validity tests whether elements believed to be unrelated to other latent variables are indeed not related. In our case, this postulate was not met because the results showed that some items were more related to different latent variables than their own. An attempt was made to improve discriminant validity by applying the procedure recommended by [67], but the results were still not favorable. Regarding this aspect, [38] achieved acceptable validity for most items on the scale by applying Aiken’s item-test validity index (2003). According to this index, “high values mean that the item measures the same as the test, while negative values indicate response coding problems or inconsistency between what the item measures and what the rest of the test measures” (p.182). We consider that this index could be a plausible alternative for calculating discriminant validity.

### Discussion of SEM analysis

The objective of the SEM analysis was to evaluate the construct validity of the SL model by analyzing the fit parameters proposed by [50]. Upon analyzing the obtained results, it was found that the complete model and its two alternate models (recursive and non-recursive) yielded very similar results. Additionally, the CFI, IFI, and RMSEA indices achieved an acceptable fit in all three models; however, the results for GFI and NFI were deficient. This indicates that our study’s results do not correspond with the results of [30, 35, 68], which reported values above 0.95 (see Table 11).

**Table 11:** Comparative studies on robustness indices from other research.

Model	X2	X2/df	GFI	NFI	CFI	IFI	RMSEA
Complete model	467.891, *p* < 0.000	1.602	0.803	0.843	0.934	0.934	0.045
Recursive model	369.914, *p* < 0.002	1.262	0.803	0.856	0.966	0.966	0.030
Non recursive model	431.532, *p* < 0.000	1.604	0.813	0.847	0.936	0.936	0.045
Espinosa et al [38]	134.83, p = 0.000	NR	0.89	0.87	0.88	0.88	0.19
Fry et al [30]	1633.29, *p* < 0.001	NR	NR	0.959	0.971	0.971	NR
Fry y Matherly [35]	2345.36, *p* < 0.001	NR	NR	0.950	0.963	0.963	NR
Fry, et al [68]	233.11, *p* < 0.001	NR	NR	0.940	0.96	0.96	0.08
Jeon, et al [34]	2010.783, *p* < 0.001	NR	NR	NR	0.908	NR	0.061
Hunsaker [36]	474.46, *p* < 0.001	NR	NR	NR	0.97	0.97	0.04
Hunsaker [37]	537.91, *p* < 0.001	NR	NR	0.905	0.955	0.956	0.05
Criteria [50]	-	< 3	> 0.90	> 0.90	> 0.90	> 0.90	< 0.08

A search for other research with results similar to ours revealed that the study [38] obtained results below 0.90. However, the validity of these results is not discussed in depth, and it is stated that these are “close to the reference values” (p. 184).

The study by [34], which validated the scale with the CFI (0.908) and RMSEA (0.061), was also examined. However, the data are too scarce to permit a comparison exercise. Finally, the studies by Hunsaker [36, 37] validated the scale using three indicators: The Comparative Fit Index (CFI), the Incremental Fit Index (IFI), and the Root Mean Square Error of Approximation (RMSEA) were utilized. The results were found to be highly comparable to those of Fry, with values indicating satisfactory adjustments in all indicators.

### Discussion of the results of the model hypotheses

When estimating the relationships between the proposed hypotheses in the complete model, it was found that all were positive and highly significant except for the relationship between vision and altruistic love (see Table 12). These results indicate that the results obtained in H1, H3, H5, and H6 were consistent with the theoretical propositions of the SL model and other studies that obtained similar results, such as [30, 33, 34, 36–38] also found positive and significant relationships in all hypotheses (see Table 12). In contrast, the results obtained in the relationship between vision and altruistic love (H2) were not significant (-0.044), and the relationship between altruistic love and vision was also not significant, with a correlation of (0.076).

**Table 12:** Comparative studies on the significance relationships between hypotheses in the complete model.

Hyp.	Relationship	Fry’s [30]	Fry’s [35]	Fry’s [33]	Jeon [34]	Hunsaker [36]	Hunsaker [37]	Espinosa [38]
H1	Hope/faith (ef) positively influences vision (vision).	0.56***	0.85***	0.67***	0.50***	0.27***	0.48***	0.44***
H2	Vision (vision) positively influences altruistic love (aa).	0.71***	0.02***	0.51***	0.49***	0.53***	0.41***	0.35***
H3	Altruistic love (aa) positively influences hope/faith (ef).	0.40***	0.44***	0.79***	0.39***	0.76***	0.59***	0.45***
H4	Altruistic love (aa) positively influences vision (vision).	0.39***	0.63***	0.27***	0.24***	0.52***	0.52***	0.17***
H5	Vision (vision) positively influences vocation (voca).	0.73***	0.77***	0.61***	0.60***	0.45***	0.58***	0.45***
H6	Altruistic love (aa) positively influences membership (belonging).	0.91***	0.92***	0.96***	0.79***	0.94***	0.97***	0.79***

Note:

*** degree of significance.

It can be postulated that the lack of significant relationships between vision and altruistic love in the context of our research may be attributed to the fact that the vision espoused by leaders does not have a sufficiently impactful effect on teachers to motivate them to engage in altruistic behaviors. Similarly, the altruistic behaviors observed by teachers in their daily work activities do not exert a notable reinforcement on the vision. Upon analysis of the response trend, it was found that the “vision” variable exhibited high scores in its inspiring capacity and clarity. However, the question “I trust the vision of my institution because it generates benefits for its employees” exhibited the lowest scores for the variable, which refers to the construction of trust as a key element in intrinsic motivation.

For example, [69] study emphasizes the importance of positive relationships between the leader and collaborators as an important factor in developing a sense of belonging. Likewise, [70] study conducted in schools states that it is imperative that teachers feel that they are admired, respected, and recognized for their contributions to the school, highlighting the importance of using motivating language. On the other hand, [34] study emphasizes the importance of leaders maintaining an alignment between individual purpose and the organizational mission. Finally, the study by [51] confirms the importance of communication and altruistic behavior as factors that improve trust and the sense of belonging.

Another potential explanation is the skepticism or sense of vulnerability that teachers may experience when providing responses that they perceive as inappropriate or inconsistent with the expectations of the organization. This is inferred from the observation that, upon review of the responses, a significant number of them fell within the options of “agree” and “neither agree nor disagree.” This could indicate that respondents chose to provide responses that they considered to be politically correct or to maintain a neutral stance. As stated by Crossman (2010), this phenomenon is frequently observed in organizations that exert pressure on individuals to participate in a spiritual culture that does not fully resonate with their “deeper self.” In this context, teachers in Adventist schools are expected to share the same religious beliefs and support the transcendent vision. However, as previously stated in hypothesis (H4), trust is “something” that is built in everyday interaction. In other words, behaviors guided by moral and spiritual values, such as cooperation, compassion, empathy, service, and altruism, help create a work environment that reinforces the vision and demonstrates consistency between beliefs and actions. In this way, the employee receives a consistent message that strengthens his or her confidence and enhances the spiritual environment of the organization.

Although the study’s results were not as expected, similarities were found with other similar studies that presented the same phenomenon. For example, [71] studied the relationship between workplace spirituality and organizational commitment. They found that there is a significant but inverse relationship between workplace spirituality and organizational commitment. According to the authors, the inverse nature of the relationship is a consequence of the negative perception employees have of current organizational policies. Similarly, other studies also showed results contrary to the preestablished theoretical postulates [72, 73]. These findings confirm that spiritual leadership is closely linked to the psychological and social aspects of the individual [2, 74]. In other words, leadership styles that work with such deep aspects of the individual, such as a sense of purpose, meaning, vocation, and transcendence, require leaders with genuine spirituality capable of inspiring and generating trust through consistency between their words and actions.

## Conclusion

According to the obtained results, the construct validity of the SL model of [35] found that the indicators showed instrument reliability and convergent validity, but not discriminant validity. Regarding the statistical robustness of the three models, it is concluded that although the indices yielded results that did not meet all previously established criteria, this does not completely invalidate the model but shows specific weaknesses. Given these weaknesses, it is considered appropriate to study in greater detail and depth the causal nature of the SL model and its recursive and non-recursive aspects. This is because the results obtained in the only reciprocal relationship of the model (vision-altruistic love). Additionally, as previously mentioned, the arguments of [63]; and [75] may be useful for explaining the obtained results. It is also concluded that empirical research with results that are not consistent with preexisting models is considered a potential opportunity to deepen and enrich the discussion in the field, both in its theoretical and methodological aspects (see example: [68, 76]). Additionally, it is considered that after nearly 20 years of validation of the SL model, this study provides new theoretical and methodological findings that open up opportunities for new avenues of research that enrich the model and sustain its practical utility in developing strategies to address the multifaceted challenges confronting contemporary organizations.

## Areas for future research

We consider that while there is a wide variety of leadership proposals based on spirituality, many of them stem from the same theoretical foundations proposed by seminal authors, suggesting a lack of new theoretical and methodological contributions. It is important to mention the increase in studies on SL contextualized in new spiritualities. The study by Makkar and Sing [77] proposes an SL model based on religions from the Asian continent and, although it maintains the same traditional theoretical logic, it adopts interesting elements of local identity. Additionally, [78] explore the utility of the “Buddhist theory of emptiness” as an alternative to cultivate an inner life that strengthens SL in managers through the development of higher levels of consciousness. It is also considered necessary to conduct research with a multilevel approach that measures the social, organizational, group, and individual impact of SL [79]. Conducting qualitative studies would also contribute to a greater understanding of the links between spirituality and contemporary organizations. Conducting cross-sectional studies that measure the impact of SL over time is another pending aspect. Finally, the relationships between spirituality and technology-mediated work environments are another topic that has not yet received sufficient attention.

## Gratitude

We appreciate the dedicated support of Professor Kenneth Roy Cabrera Torres, Geologist, Systems Engineer, Master in Statistics, and Full-Time Professor at the Laboratory of Complex Natural Systems, Faculty of Sciences, Universidad Nacional de Colombia, Campus Medellín, who served as the statistical advisor for this research. His guidance and support were crucial for carrying out the study.

## Supporting information

S1 File Fry and Matherly’s (2006) spiritual leadership questionnaire (SLT). (DOCX)

S2 File Cuestionario de Liderazgo espiritual (SLT) de Fry y Matherly (2006). (DOCX)

S3 File (RAR)

S1 Data (XLSX)
