# Supplementary material for: Revisiting Louis Fry’s spiritual leadership model in confessional school teachers using structural equation modeling (SEM)
Source: PLoS One. 2024 Sep 17;19(9):e0299671. doi: 10.1371/journal.pone.0299671 (PMC11407629; doi:10.1371/journal.pone.0299671)
Supplement: S1 File — (DOCX) [file pone.0299671.s001.docx]

Fry and Matherly's (2006) Spiritual Leadership Questionnaire (SLT)

1. Completely disagree
2. Disagree
3. No opinion
4. Agree
5. Strongly agree

| N. | Category | Sub-category | Question | 1 | 2 | 3 | 4 | 5 |
| --- | --- | --- | --- | --- | --- | --- | --- | --- |
| 1 | Spiritual leadership (Fry y Matherly, 2006) | Visión | I understand and am committed to my organization’s vision. |  |  |  |  |  |
| 2 |  |  | My workgroup has a vision statement that brings out the best in me. |  |  |  |  |  |
| 3 |  |  | My organization’s vision inspires my best performance. |  |  |  |  |  |
| 4 |  |  | I have faith in my organization’s vision for its employees. |  |  |  |  |  |
| 5 |  |  | My organization’s vision is clear and compelling to me. |  |  |  |  |  |
| 6 |  | Hope/faith | I have faith in my organization and I am willing to bdo whatever it takes to insure that it accomplishes its mission. |  |  |  |  |  |
| 7 |  |  | I persevere and exert extra effort to help my organization succeed because I have faith in what it stands for. |  |  |  |  |  |
| 8 |  |  | I always do my best in my work because I have faith in my organization and its leaders. |  |  |  |  |  |
| 9 |  |  | I set challenging goals for my work because I have faith in my organization and want us to succeed. |  |  |  |  |  |
| 10 |  |  | I demonstrate my faith in my organization and its mission by doing everything I can to help us succeed. |  |  |  |  |  |
| 11 |  | Altruistic love | My organization really cares about its people. |  |  |  |  |  |
| 12 |  |  | My organization is kind and considerate toward its workers, and when they are suffering, wants to do something  about it. |  |  |  |  |  |
| 13 |  |  | The leaders in my organization walk the walk as well as talk the talk. |  |  |  |  |  |
| 14 |  |  | My organization is trustworthy and loyal to its employees. |  |  |  |  |  |
| 15 |  |  | My organization does not punish honest mistakes. |  |  |  |  |  |
| 16 |  |  | The leaders in my organization are honest and without false pride |  |  |  |  |  |
| 17 |  |  | The leaders in my organization have the courage to stand up for their people. |  |  |  |  |  |
| 18 | Spiritual well-being (Fry y Nisieiwcz, 2013). | Meaning/calling | The work I do is very important to me. |  |  |  |  |  |
| 19 |  |  | My job activities are personally meaningful to me. |  |  |  |  |  |
| 20 |  |  | The work I do is meaningful to me. |  |  |  |  |  |
| 21 |  |  | The work I do makes a difference in people’s lives. |  |  |  |  |  |
| 22 |  | Membership | I feel my organization understands my concerns. |  |  |  |  |  |
| 23 |  |  | I feel my organization appreciates me, and my work. |  |  |  |  |  |
| 24 |  |  | I feel highly regarded by my leadership. |  |  |  |  |  |
| 25 |  |  | I feel I am valued as a person in my job. |  |  |  |  |  |
| 26 |  |  | I feel my organization demonstrates respect for me, and my work. |  |  |  |  |  |
