# Supplementary material for: Revisiting Louis Fry’s spiritual leadership model in confessional school teachers using structural equation modeling (SEM)
Source: PLoS One. 2024 Sep 17;19(9):e0299671. doi: 10.1371/journal.pone.0299671 (PMC11407629; doi:10.1371/journal.pone.0299671)
Supplement: S2 File — (DOCX) [file pone.0299671.s002.docx]

Cuestionario de Liderazgo espiritual (SLT) de Fry y Matherly (2006)

1. Completamente desacuerdo
2. En desacuerdo
3. No opino
4. De acuerdo
5. Completamente de acuerdo

| N. | Categoría | Sub-categoría | Pregunta | 1 | 2 | 3 | 4 | 5 |
| --- | --- | --- | --- | --- | --- | --- | --- | --- |
| 1 | Liderazgo espiritual (Fry y Matherly, 2006) | Visión | Comprendo la visión de mi organización y estoy comprometido con ella. |  |  |  |  |  |
| 2 |  |  | Mi grupo de trabajo tiene una declaración de visión que saca lo mejor de mí. |  |  |  |  |  |
| 3 |  |  | La visión de mi organización me inspira a dar lo mejor de mí en lo que hago. |  |  |  |  |  |
| 4 |  |  | Tengo fe en la visión de mi organización porque genera beneficios para sus empleados. |  |  |  |  |  |
| 5 |  |  | La visión de mi organización es clara, apremiante y me impulsa. |  |  |  |  |  |
| 6 |  | Esperanza/fe | Tengo fe en mi organización y estoy dispuesto a hacer lo que sea necesario para garantizar que se cumpla su misión. |  |  |  |  |  |
| 7 |  |  | Persevero y llevo a cabo esfuerzos adicionales para ayudar a mi organización a que tenga éxito porque tengo fe en lo que representa. |  |  |  |  |  |
| 8 |  |  | Siempre doy lo mejor de mí en mi trabajo porque tengo fe en mi organización y sus líderes. |  |  |  |  |  |
| 9 |  |  | Establezco objetivos desafiantes en mi trabajo porque tengo fe en mi organización y quiero que ambos tengamos éxito. |  |  |  |  |  |
| 10 |  |  | Manifiesto fe en mi organización y su misión haciendo todo lo que está a mi alcance para ayudarle a ella y a mí a alcanzar el éxito. |  |  |  |  |  |
| 11 |  | Amor altruista | Mi organización realmente se preocupa por su gente. |  |  |  |  |  |
| 12 |  |  | Mi organización es amable y considerada con sus trabajadores y cuando sufren quiere hacer algo al respecto. |  |  |  |  |  |
| 13 |  |  | Los líderes en mi organización respaldan sus palabras con hechos, o sea, son capaces de llevar a cabo lo que dicen, no son solo palabras. |  |  |  |  |  |
| 14 |  |  | Mi organización es digna de confianza y leal con sus empleados. |  |  |  |  |  |
| 15 |  |  | Mi organización no castiga los errores cometidos sin intención y honestamente. |  |  |  |  |  |
| 16 |  |  | Los líderes en mi organización son honestos y sin falso orgullo. |  |  |  |  |  |
| 17 |  |  | Los líderes en mi organización tienen el valor de defender a su gente. |  |  |  |  |  |
| 18 | Bienestar espiritual (Fry y Nisieiwcz, 2013). | Significado/vocación | El trabajo que yo hago es muy importante para mí. |  |  |  |  |  |
| 19 |  |  | Las actividades que llevo a cabo en mi trabajo son significativas para mí desde el ámbito personal. |  |  |  |  |  |
| 20 |  |  | El trabajo que hago es significativo para mí. |  |  |  |  |  |
| 21 |  |  | El trabajo que hago hace una diferencia en la vida de las personas. |  |  |  |  |  |
| 22 |  | Pertenencia/membresía | Siento que mi organización comprende mis preocupaciones. |  |  |  |  |  |
| 23 |  |  | Siento que mi organización me aprecia a mí y mi trabajo. |  |  |  |  |  |
| 24 |  |  | Siento que mis líderes me aprecian mucho. |  |  |  |  |  |
| 25 |  |  | Siento que soy valorado como persona en mi trabajo. |  |  |  |  |  |
| 26 |  |  | Siento que mi organización demuestra respeto por mí y mi trabajo. |  |  |  |  |  |
